# 晚期非小细胞肺癌RRM1蛋白表达与吉西他滨联合顺铂化疗疗效的关系

**DOI:** 10.3779/j.issn.1009-3419.2011.04.07

**Published:** 2011-04-20

**Authors:** 志强 高, 宝惠 韩, 洁 沈, 爱琴 顾, 华 钟

**Affiliations:** 200030 上海，上海交通大学附属胸科医院肺内科 Department of Pulmonary Medcine, Chest Hospital Afliated to Shanghai Jiao Tong University, Shanghai 200030, China

**Keywords:** 肺肿瘤, RRM1, 蛋白表达, 疗效, 预后, Lung neoplasms, RRM1, Protein expression, Curative effect, Prognosis

## Abstract

**背景与目的:**

核糖核苷酸还原酶M1（ribonucleotide reductase M1, RRM1）的表达水平与肿瘤细胞对吉西他滨耐药密切相关。本研究旨在探讨晚期非小细胞肺癌（non-small cell lung cancer, NSCLC）中RRM1蛋白的表达水平与吉西他滨联合顺铂（GP方案）化疗疗效的关系。

**方法:**

应用免疫组织化学染色法检测75例晚期NSCLC组织中的RRM1蛋白表达，75例患者均接受GP化疗方案，回顾调查患者的一般特征、治疗反应、疗效评价及生存时间。组间差异采用卡方检验，采用*Kaplan-Meier*法进行生存分析。

**结果:**

RRM1蛋白表达阳性率为38.7%，与患者的性别、年龄、吸烟状态、临床分期及组织病理学类型无明显相关性（*P* > 0.05）；RRM1蛋白高表达组的化疗有效率（31.1%）低于低表达组（41.3%），有统计学意义（*P*=0.005）；RRM1高表达组的1年生存率（27.6%）低于低表达组（58.7%），有统计学意义（*P*=0.009）；RRM1蛋白高表达组的中位生存期（10.70个月）低于低表达组（13.30个月），但无统计学差异（*P*=0.245）；RRM1蛋白高表达组的疾病进展时间（3.10个月）低于低表达组（5.11个月），差异有统计学意义（*P*=0.042）。

**结论:**

晚期NSCLC患者组织中RRM1蛋白的表达水平与GP方案化疗的疗效及预后密切相关。

## Abstract (en)

**Background and objective:**

It has been proven that ribonucleotide reductase M1 (RRM1) expression level was closely related to gemcitabine drug-resistance to tumor cells. Te aim of this study is to explore the relations between RRM1 protein expression levels and effects of gemcitabine and cisplatin chemotherapy in advanced non-small cell lung cancer (NSCLC) patients.

**Methods:**

The expressions of RRM1 in 75 advanced NSCLC tissues were qualitatively detected by immunohistochemical methods. Seventy-fve patients had received gemcitabine and cisplatin (GP) chemotherapy regimen. General characteristics of patients, response to treatment, efcacy evaluation and survival time were retrospectively investigated. Differences between the groups were statistically analysed by chi-square test. Survival differences were analysed by temporal inspection and *Kaplan-Meier* survival curves.

**Results:**

RRM1 protein expression rate was 38.7%. RRM1 protein expression had no obvious relationship with gender, age, smoking status, and the clinical stages and histopathological types (*P* > 0.05). Response rate in RRM1 protein high expression groups (31.3%) was remarkably lower than that in low expression groups (41.3%), the difference was statistically signifcant (*P*=0.005). 1-year survival rate in RRM1 protein high expression groups (27.6%) was remarkably lower than that in low expression group (58.7%), the difference was statistically signifcant (*P*=0.009). No signifcant different of median survival was observed between RMM1 protein high expression group and low expression group (*P* > 0.245). Time to progression in RRM1 protein high expression groups (3.10 months) was remarkably lower than that in low expression group (5.11 months), the difference was statistically signifcant (*P*=0.042).

**Conclusion:**

RRM1 protein expression levels are closely related to effects of gemcitabine and cisplatin chemotherapy and prognosis of advanced NSCLC patients.

肺癌是全球发病率和病死率最高的肿瘤，其中约80%为非小细胞肺癌（non-small cell lung cancer, NSCLC）。因缺乏有效的早期诊断手段，初诊时约75%的患者已失去了手术时机，晚期NSCLC目前仍以联合化疗为主，但5年生存率不到15%^[1]^。影响NSCLC患者化疗疗效及生存的主要原因是肿瘤细胞对抗癌药的抗药性。核糖核苷酸还原酶M1（ribonucleotide reductase M1, RRM1）是最近被广泛关注的一个基因，RRM1的表达水平与肿瘤细胞对吉西他滨耐药密切相关^[2]^。通过对RRM1表达的检测，能否预测不同人群对吉西他滨的敏感程度以制定个体化化疗方案、提高药物治疗的有效率，这些都成为人们感兴趣和研究的主要目标。然而，近几年国内的相关研究均未得到理想的试验结果。在国内外研究的基础上，本研究探讨了RRM1蛋白的表达水平与吉西他滨联合顺铂（GP方案）治疗晚期NSCLC疗效的关系。

## 1 材料与方法

### 1.1 研究对象

以2006年8月-2009年5月上海市胸科医院收治的既往未接受过化疗、无法手术切除的75例晚期（Ⅲb期-Ⅳ期）NSCLC患者为研究对象，75例患者均经支气管镜下活检（58例）或穿刺活检（17例）获得病理学诊断。其中男性50例，女性25例；年龄35岁-73岁，中位年龄60岁；肺鳞癌21例，肺腺癌49例，其他类型5例。全部患者均具有可测量的肿瘤病灶，参照国际抗癌联盟1997年TNM分期，Ⅲb期34例，Ⅳ期41例。吸烟者47例，不吸烟者28例。75例患者均接受GP方案（吉西他滨1, 000 mg/m^2^ d1，d 8+顺铂75 mg/m^2^ d1，每28天为1周期）。末次随访时间为2010年9月30日。

### 1.2 主要试剂

鼠抗人RRM1单克隆抗体购自Santa Cruz生物工程公司，免疫组织化学试剂盒购自华美生物工程公司。

### 1.3 实验方法

收集75例NSCLC纤支镜或肺穿刺活检标本，用微波行抗原修复15 min，鼠抗人RRM1单克隆抗体（1:100）4 ℃过夜，磷酸盐缓冲液（PBS, pH7.2）清洗后加Anti-Rabbit Envision HRP二抗37 ℃放置30 min，PBS清洗后DAB显色，苏木精对比染色，然后水洗、蓝化、脱水、中性树胶封片。以Lecia QWin Plus软件对组织芯片结果进行分析，RRM1主要为胞浆染色，胞浆中出现棕黄色颗粒且面积占分析区域总面积比≥5%为RRM1（+）表达即高表达， < 5%为RRMl（-）即低表达^[3]^。由病理科两位医师（其中一位为主任医师）在不知道临床资料的情况下对标本染色结果进行判断。

### 1.4 疗效评价标准

按世界卫生组织与国际抗癌联盟标准判定为完全缓解（complete response, CR）、部分缓解（partial response, PR）、轻度缓解（minimal response, MR）、疾病稳定（stable disease, SD）与疾病进展（progressive disease, PD）。近期客观有效为CR+PR+MR，受益为CR+PR+MR+SD，有效者应1个月后再检查确认。疾病进展时间（time to progress, TTP）为自治疗开始至肿瘤病灶出现进展的时间。生存期为治疗开始至死亡或失访的时间。行为状态采用东部肿瘤协作组的体能状态评分（Eastern Cooperative Oncology Group Performance Status Scale, ECOG-PS）。

### 1.5 统计学分析

采用SPSS 13.0软件进行数据分析。组间差异采用卡方检验。生存分析采用*Kaplan-Meier*法，生存率的比较采用*Log-rank*检验，*P* < 0.05为差异有统计学意义。

## 2 结果

### 2.1 RRM1蛋白在晚期NSCLC组织中的表达及其与临床特征之间关系

75例初治晚期NSCLC患者的病理标本中，RRM1蛋白表达阳性率为38.7%，与患者的性别、年龄、吸烟状态、临床分期及组织病理学类型无明显相关性（*P* > 0.05）（图 1，表 1）。

**1:**
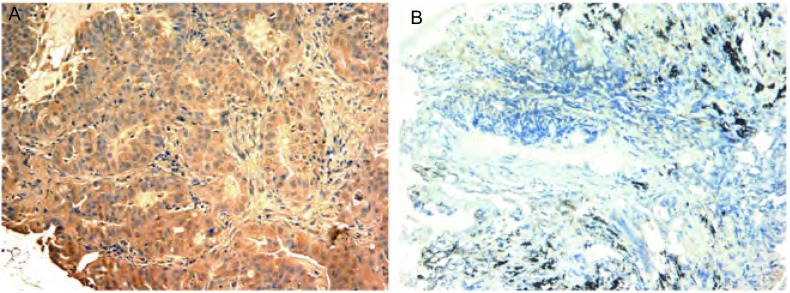
RRM1蛋白在晚期NSCLC组织中的表达（SP，×200）。A：阳性表达；B：阴性表达。 Expression of RRM1 in advanced NSCLC tissues (SP, ×200). A: Positive; B: Negative.

**1:** 各组晚期NSCLC患者的临床特征 Characteristics of patients with advanced NSCLC

Characteristic	All patients			RRM1(-) subgroup			RRM1(+) subgroup	
	*n*	%		*n*	%		*n*	%
Total (*n*)	75			46			29	
Sex								
Male	50	66.7		31	67.4		19	65.5
Female	25	33.3		15	32.6		10	34.5
Smoking Status								
Non-smoker	28	37.3		18	39.1		10	34.5
Smoker	47	62.7		28	60.9		19	65.5
Age (years)								
Median	60			61			59	
Range	35-73			38-71			35-73	
ECOG PS								
0	38	50.7		21	53.2		17	55.8
1	37	49.3		25	46.8		12	44.2
Histology								
Adenocarcinoma	49	65.3		31	67.4		18	62.1
Squamous cell carcinoma	21	28.0		12	26.1		9	31.0
Others	5	6.7		3	6.5		2	6.9
Stage								
Ⅲb	34	45.3		21	41.8		13	44.2
Ⅳ	41	57.4		25	58.2		16	55.8
No. of cycles								
Median	4			4			4	
Range	2-15			2-10			2-15	
ECOG PS: Eastern Cooperative Oncology Group Performance Status Scale.								

### 2.2 RRM1蛋白表达与化疗疗效及生存期之间的关系

RRM1蛋白高表达组的化疗有效率（31.1%）低于低表达组（41.3%），差异有统计学意义（χ^2^=10.558, *P*=0.005）；RRM1表达阳性组的1年生存率（27.6%）低于阴性组（58.7%），差异有统计学意义（χ^2^=6.916, *P*=0.009）；RRM1蛋白高表达组的中位生存期（10.70个月，95%CI：7.91-13.49）低于低表达组（13.30个月，95%CI：11.61-14.94），但差异无统计学意义（*P*=0.245）；RRM1蛋白高表达组的疾病进展时间为（3.10个月，95%CI：1.65-4.55）低于低表达组（5.11个月，95%CI：3.58-6.42），差异有统计学意义（*P*=0.042）（图 2，表 2）。

**2:**
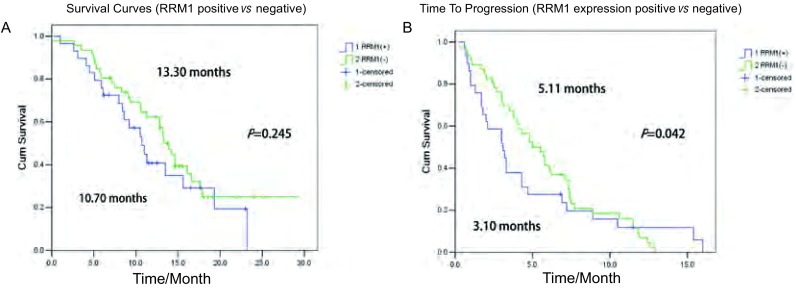
RRM1表达水平与NSCLC患者的生存曲线之间的关系。A：总体生存时间；B：疾病进展时间。 Relationships between expressions of RRM1 and the survival curves of NSCLC patients. A: overall survival; B: time to progression.

**2:** 晚期NSCLC患者的临床疗效及预后 Response to treatment and survival time of patients with advanced NSCLC

Outcome	All patients (*n*=75)		RRM1 (-) subgroup (*n*=46)		RRM1 (+) subgroup (*n*=29)	
Response						
Complete response [*n* (%)]	0		0		0	
Partial response [*n* (%)]	28 (37.3%)		19(41.3%)		9(31.1%)	
Stable disease [*n* (%)]	24 (32.0%)		19(41.3%)		5 (17.2%)	
Progressive disease [*n* (%)]	23 (30.7%)		8 (17.4%)		15(51.7%)	
Survival						
Median (months)	13.20	95%CI: 10.92-15.48	13.30	95%CI: 11.66-14.94	10.70	95%CI: 7.91-13.49
1 year survival rate (%)	46.70	95%CI: 36.40-53.20	58.70	95%CI: 48.50-68.30	27.60	95%CI: 22.40-38.10
Median TTP (months)	4.30	95%CI: 3.20-5.40	5.11	95%CI: 3.58-6.42	3.10	95%CI: 1.65-4.55
TTP: time to progress.							

## 3 讨论

近年研究^[4]^表明，细胞信号转导中相关因子的表达异常、肿瘤细胞DNA修复的异常及其它相关基因的表达异常与肺癌耐药的产生也存在密切联系，提示前瞻性地进行分子指标的测定是进行个体化治疗、提高化疗疗效的关键。

RRM1是核苷酸还原酶调节M1亚单位，当ERCC1与*XPD*、*XPG*、*XPA*等修复基因将DNA链中受损的部分切除后，DNA链上留下的空缺就由RRM1提供的核苷酸来填补^[5]^。目前研究表明，RRM1是导致肿瘤细胞对吉西他滨类耐药的一个重要因素。

Rosell等^[6]^采用定量PCR的方法检测了75例NSCLC患者的肿瘤标本中RRM1 mRNA等的表达，其中有22例患者接受了GP方案化疗，发现低水平表达RRM1 mRNA的吉西他滨联合顺铂组患者对化疗的反应较好。Lee等^[7]^研究以免疫组织化学法检测了40例晚期NSCLC组织中RRM1蛋白的表达，结果发现RRM1蛋白表达阳性率为35%，RRM1蛋白阳性表达组患者的疾病控制率（PR+SD）低于阴性表达组患者（23% *vs* 56%）。本研究显示RRM1蛋白表达阳性率为38.7%，RRM1蛋白高表达组的化疗有效率（31.1%）低于低表达组（41.3%），差异有统计学意义（*P*=0.005）；RRM1蛋白高表达组的1年生存率（27.6%）低于低表达组（58.7%），差异有统计学意义（*P*=0.009）。

Ceppi等^[8]^检测了70例NSCLC患者组织中RRM1 mRNA等的表达水平，结果发现低表达RRM1 mRNA的患者的中位生存时间较长（13.9个月*vs* 10.9个月，*P*=0.039)。Lee等^[7]^研究发现RRM1蛋白阳性表达组患者的总体生存期明显短于阴性表达组患者（5.1个月*vs* 12.9个月，*P*=0.022）。本研究显示RRM1蛋白高表达组的中位生存期（10.70个月）低于低表达组（13.30个月），但差异无统计学意义（*P*=0.245）；RMM1蛋白高表达组的疾病进展时间为（3.10个月）低于低表达组（5.11个月），差异有统计学意义（*P*=0.042）。

通过本研究进一步证实，在晚期化疗的NSCLC患者中RRM1蛋白的表达水平与肿瘤细胞对吉西他滨耐药及患者预后密切相关，提示RRM1蛋白表达水平高低可能会成为筛选接受化疗的患者的一项指标。通过对RRM1蛋白的检测，可以预测不同人群对吉西他滨的敏感程度以指导临床用药、提高药物治疗的有效率，同时减少不必要的药物毒副作用和经济支出，更好地实现对患者的个体化治疗。

总之，RRM1蛋白的表达水平与接受GP方案治疗的晚期NSCLC患者的化疗疗效及预后密切相关。但RRM1蛋白能否作为筛选患者的指标并真正应用于临床，尚有许多重要问题需要解决。
